# Building an Age-Friendly Workforce by Embedding the 4Ms Framework in a Geriatrics Clerkship

**DOI:** 10.1093/geroni/igaf122.2942

**Published:** 2025-12-31

**Authors:** Lenny Powell, Jennifer DeGennaro, Christian White, Kevin Overbeck

**Affiliations:** Rowan-Virtua School of Osteopathic Medicine, Stratford, New Jersey, United States; Rowan-Virtua School of Osteopathic Medicine, Stratford, New Jersey, United States; Rowan-Virtua School of Osteopathic Medicine, Stratford, New Jersey, United States; Rowan-Virtua School of Osteopathic Medicine, Stratford, New Jersey, United States

## Abstract

Rowan-Virtua School of Osteopathic Medicine (RVSOM) Department of Geriatrics and Gerontology embedded the 4Ms framework (What Matters, Medication, Mentation, Mobility) into the required 4-week Geriatrics Clerkship curriculum. Students participate in a full day of in-person didactics delivered by an interdisciplinary team of faculty during the first week of the clerkship designed to teach them to use evidence-based screening tools and employ best practices when caring for older patients. A survey on the 4Ms approach is distributed at the beginning of the rotation and again at the end of the 4 weeks. Pre- and post-clerkship data for 184 students show significant differences on all but 1 of 16 items [e.g., knowledge to perform a Geriatric Assessment: t(183) = 19.32, p = <.001, d = 1.42, identifying inappropriate medications: t(183) = -18.51, p = <.001, d = -1.36, and initiating an Advance Care Planning conversation: t(182) = -13.57, p = <.001, d = -1.03]. In the post survey, students are asked to specify any new practices in their care of patients after the Geriatrics clerkship and over half (54%) reported new care practices. Commonly noted were increased attention to medication lists, functional history, goals of care, and use of evidence-based screening tools. The statement “Knowing what questions to ask geriatrics pts even when I’m in another specialty” illustrates that providing foundational knowledge on aging and training students to assess patients using the 4Ms Framework creates a healthcare workforce able to recognize key issues critical to caring for an aging population.

